# The future of cancer immunotherapy for brain tumors: a collaborative workshop

**DOI:** 10.1186/s12967-022-03438-z

**Published:** 2022-05-23

**Authors:** Christine E. Brown, Samantha Bucktrout, Lisa H. Butterfield, Olga Futer, Evanthia Galanis, Adilia Hormigo, Michael Lim, Hideho Okada, Robert Prins, Sara Siebel Marr, Kirk Tanner

**Affiliations:** 1 City of Hope National Medical Center, Duarte, CA 91010 USA; 2 Parker Institute for Cancer Immunotherapy, 1 Letterman Dr. D3500, San Francisco, CA 94129 USA; 3 National Brain Tumor Society, Newton, MA 02458 USA; 4 Mayo Clinic, Rochester, MN 55905 USA; 5 Icahn School of Medicine at Mount Sinai, The Tisch Cancer Institute, New York, NY 10029 USA; 6 Stanford University School of Medicine, Stanford, CA 94305 USA; 7 Department of Neurosurgery, University of California San Francisco, San Francisco, CA 94158 USA; 8 Department of Neurosurgery and Molecular & Medical Pharmacology, David Geffen School of Medicine at UCLA, Los Angeles, CA 90095 USA; 9 Present Address: Centivax, Inc, South San Francisco, CA 94080 USA

**Keywords:** Brain tumors, Immunotherapy, Cell therapy

## Abstract

Harnessing the effector mechanisms of the immune system to combat brain tumors with antigen specificity and memory has been in research and clinical testing for many years. Government grant mechanisms and non-profit organizations have supported many innovative projects and trials while biotech companies have invested in the development of needed tools, assays and novel clinical approaches. The National Brain Tumor Society and the Parker Institute for Cancer Immunotherapy partnered to host a workshop to share recent data, ideas and identify both hurdles and new opportunities for harnessing immunotherapy against pediatric and adult brain tumors. Adoptively transferred cell therapies have recently shown promising early clinical results. Local cell delivery to the brain, new antigen targets and innovative engineering approaches are poised for testing in a new generation of clinical trials. Although several such advances have been made, several obstacles remain for the successful application of immunotherapies for brain tumors, including the need for more representative animal models that can better foreshadow human trial outcomes. Tumor and tumor microenvironment biopsies with multiomic analysis are critical to understand mechanisms of response and patient stratification, yet brain tumors are especially challenging for such biopsy collection. These workshop proceedings and commentary shed light on the status of immunotherapy in pediatric and adult brain tumor patients, including current research as well as opportunities for improving future efforts to bring immunotherapy to the forefront in the management of brain tumors.

## Introduction

The National Brain Tumor Society (NBTS) and the Parker Institute for Cancer Immunotherapy (PICI) convened a group of experts for focused discussions about advancing the impact of immuno-oncology (IO) in brain tumors (Table 1). The workshop focused on 3 critical questions: Where are we in the journey to make IO effective in brain tumors? What is most promising that should be emphasized? What can be done to improve the future for IO in brain tumor research and treatment development? We present a brief state of the field, critical hurdles impeding progress, promising new technologies, and several proposed approaches in the field of IO for brain tumors. A thorough review of IO in brain tumors has been recently published [1] and will not be repeated here.

**Table 1:** Workshop participants

Name	Affiliation	Location
David Anderson	VBI	MA
David Andrews, MD	Imvax	PA
Terri Armstrong, PhD, ANP-BC, FAAN, FAANP	National Institutes of Health Intramural Research Program	MD
David Arons, JD	National Brain Tumor Society	MA
Amy Barone, MD	US Food and Drug Administration	MD
Diana Bradford, MD	US Food and Drug Administration	MD
Christine Brown, PhD	City of Hope	CA
Samantha Bucktrout, PhD	Parker Institute for Cancer Immunotherapy	CA
Alexandra Butler, MD	Patient Advocate	OR
Lisa Butterfield, PhD	Parker Institute for Cancer Immunotherapy	CA
Ari Britton, MSc	Parker Institute for Cancer Immunotherapy	CA
Yan Chen, PhD	Elpis Biopharmaceuticals	MA
Tim Cloughesy, MD	University of California Los Angeles	CA
Bob Dillman, MD	AIVITA Biomedical	CA
Ute Dugan, MD, PhD	Parker Institute for Cancer Immunotherapy	CA
Brett Ewald, PhD	DNAtrix	CA
Justin Fairchild, MPH	Parker Institute for Cancer Immunotherapy	CA
Olga Futer, PhD, PMP	National Brain Tumor Society	MA
Evanthia Galanis, MD	Mayo Clinic	MN
Katie Germain	National Brain Tumor Society	MA
Dan Getts, MBA, PhD	Myeloid Therapeutics	MA
Pier Federico Gherardini, PhD	Parker Institute for Cancer Immunotherapy	CA
Mark Gilbert, MD	National Institutes of Health Intramural Research Program	MD
Sangeeta Goswami, MD, PhD	MD Anderson Cancer Center	TX
Natalie Greco, PhD	Parker Institute for Cancer Immunotherapy	CA
Melinda Griffith, JD	Parker Institute for Cancer Immunotherapy	CA
Thomas Halkin	National Brain Tumor Society	MA
Jen Haslip	Parker Institute for Cancer Immunotherapy	CA
Amy Heimberger, MD	Northwestern Medicine	IL
Adilia Hormigo, MD, PhD	Mount Sinai	NY
John Infanti, MPA	Parker Institute for Cancer Immunotherapy	CA
Hilary Keeley, JD	The Sontag Foundation	FL
Lacey Kitch, PhD	Parker Institute for Cancer Immunotherapy	CA
Theresa LaVallee, PhD	Parker Institute for Cancer Immunotherapy	CA
Barbara Lavery	ACGT Foundation	CT
Danielle Leach, MPH	National Brain Tumor Society	MA
Michael Lim, MD –	Stanford Medicine	CA
Wendell Lim, PhD	University of California San Francisco	CA
Sara Siebel Marr, MS	Parker Institute for Cancer Immunotherapy	CA
Clair Meehan, MEM	National Brain Tumor Society	MA
Duane Mitchell, MD, PhD	University of Florida	FL
W. Garrett Nichols, MD, MS	Istari Oncology	NC
Hideho Okada, MD, PhD	University of California San Francisco	CA
Derek Oldridge, MD, PhD	University of Pennsylvania	PA
Donald O’Rourke, MD	University of Pennsylvania	PA
Jeremy Pivor	Patient Advocate	MA
Janine Pixley, MFA, CFRE	Parker Institute for Cancer Immunotherapy	CA
Robert Prins, PhD	University of California Los Angeles	CA
Raj Puri, MD, PhD	US Food and Drug Administration	MD
Christophe Quéva, PhD	Oncorus	MA
David Reardon, MD	Dana-Farber Cancer Institute	MA
Joan Robbins, PhD	DNAtrix	CA
Kole Roybal, PhD	University of California San Francisco	CA
Daniel Ryan	The Sontag Foundation	FL
Wendy Selig	WSCollaborative	VA
Jeffrey Skolnik, MD	Inovio Pharmaceuticals	CA
William Smith, PhD, JD	Parker Institute for Cancer Immunotherapy	CA
Jessica Sorrentino, PhD	Istari Oncology	NC
Marko Spasic, MD	Parker Institute for Cancer Immunotherapy	CA
Tresa Spencer, PhD	Patient Advocate	TX
Katherine Szarama, PhD	Emerson Collective	CA
Kirk Tanner, PhD	National Brain Tumor Society	MA
Reena Thomas, MD, PhD	Stanford Medicine	CA
William Timmer, PhD	National Cancer Institute	MD
Leo Wang, MD, PhD	City of Hope	CA
Patrick Wen, MD	Dana-Farber Cancer Institute	MA
Jingying Xu, PhD	Parker Institute for Cancer Immunotherapy	CA
Alfred Yung, MD	MD Anderson Cancer Center	TX

## What is the state of IO for brain tumors in academia and nonprofits?

Primary brain tumors, such as glioblastoma (GBM), have been remarkably resistant to immunotherapy, even though preclinical models suggest effectiveness. While immune checkpoint blockade molecules, notably targeting the PD-1 / PD-L1 pathways, have revolutionized treatment of other tumor types, brain tumors have shown limited efficacy in the adjuvant setting [2], although the neoadjuvant setting has shown some promise [3]. In other cancer types, characterization of the immune composition of the tumor microenvironment has revealed significant heterogeneity across tumor subtypes and patients, with high diversity in the intratumoral compartments. In certain cases, unique novel lymphoid or myeloid subsets emerge within the tumor that are absent from adjacent normal tissue or peripheral blood, and such specific immune compositions may affect response and resistance to therapy and overall survival. The brain has been characterized as a immuneprivelged organ, with multiple mechanisms of limiting potentially destructive immune responses. Thus, the brain may present multiple, inherent resistance mechansims to productive immunotherapy. In the GBM tumor microenvironment (TME), myeloid cells comprise a significant proportion of immune cells. In addition to inducing T-cell infiltration into brain tumors, immunotherapy may create a cascade of pro-inflammatory events in the CNS that are counter-regulated by an induced immigration of immunosuppressive myeloid cells to the GBM TME. These cells are phenotypically similar to the myeloid cells that dominantly attenuate the T-cell response to chronic viral infections and may counteract the effective anti-tumor T-cell responses induced by dendritic cell (DC) vaccination or other systemic immune-stimulalatory therapies within the tumor microenvironment. As such, myeloid cells may mediate adaptive immune resistance in response to T-cell activation induced by immunotherapy. A better understanding of the biology of these cells and the mechanisms by which this cell population negatively regulates the anti-tumor immune response could be critical to inducing effective anti-tumor immune responses in these malignant brain tumors.

Because rational drug and clinical trial design in high-grade gliomas will be biomarker-driven, recent efforts have focused on generating relevant datasets for clinical and drug development decision-making. PICI has partnered with Cancer Research Institute (CRI) and American Cancer Gene Therapy (ACGT) non-profit organizations in a consortia effort to demystify heterogeneous expression of known immunogenic glial targets, and the quantity, quality and spatial characteristics of immune cells and immune-therapeutic targets across a large number of high-grade glioma samples, many of which are from patients involved in immunotherapy clinical trials. To generate a ground truth of biological signatures across the datasets, transcriptomic, genomic and protein data from assays that have provided unique insights into the biology and prognosis of gliomas [4–8] will be integrated. Single-cell transcriptomics will provide insights into immune cell infiltrate, whereas bulk transcriptomics captures data from the stroma and tumor that get lost during tissue dissociation to generate single cells. Deeper insights into tumor and immune cell interactions and phenotypes in intact glial tissues using high dimensional immune profiling and high resolution protein expression mapping [8] may reveal biological networks that translate to biomarkers to guide therapeutic decision making [7]. Such multi-modal atlases will provide invaluable datasets for the field to accelerate research and development advances in gliomas and other CNS tumors.

In addition to facilitation a comprehensive evaluation of the immune microenvironment, partnership efforts between academia and non-profits have also focused on generating novel therapeutics. One area of intense intersest include chimeric antigen receptor (CAR) T cell therapies (Table 2). To generate de novo antitumor T cell responses against GBM and other primary adult brain tumors, several groups are clinically evaluating chimeric antigen receptor (CAR) T cell therapies to target tumor-associated antigens (TAAs) commonly expressed on the surface of these malignant tumors, but not normal brain tissue. City of Hope was the first to clinically translate CAR T cells for the treatment of brain tumors and has pioneered the locoregional delivery of CAR T cells, in which the therapeutic cells are delivered via a reservoir (Ommaya)/catheter device into either the tumor bed, tumor resection cavity or cerebrospinal fluid. Preclinical studies have established that, based on cell dose, locoregional delivery is more effective than systemic delivery for these regionally restricted central nervous system (CNS) tumors [9–11] Further, the catheter/reservoir allows for local sampling of the cerebrospinal fluid (CSF) and tumor fluid during treatment, thereby providing critical insights into the local CNS response to therapy [12]. Current lead clinical programs are evaluating the locoregional delivery of CAR T cells targeting the glioma TAA interleukin-13 receptor (IL13R)α2, a high-affinity IL13 receptor, and has open trials for IL13Rα2-CAR as a single agent therapy for recurrent high-grade glioma [9, 12] (NCT02208362) and other primary brain tumors (NCT04661384) [13] in combination with checkpoint inhibition [14] (NCT04003649; or with added lymphodepletion (NCT04510051). With the goal of expanding the repertoire of targets amenable for brain tumor CAR T cell therapy, this group is also evaluating HER2-targeted CAR T cells in the recurrent GBM setting (NCT03389230) as well as HER2 + breast cancer that has metastasized to the brain (NCT03696030). Chlorotoxin (CLTX)-peptide directed CAR T cells are also being tested in the recurrent GBM setting (NCT04214392). The CLTX-CAR harnesses the ability of the scorpion venom derived CLTX peptide to selectively bind a receptor on GBM that involves MMP-2 but not normal brain tissue [15].

**Table 2:** Current Pediatric CAR-T Cell Therapy Clinical Trials

Antigen target	Cellular product	Key inclusion	NCT number
GD2	CAR-T	H3K27M-mutant diffuse intrinsic pontine glioma (DIPG) or spinal H3K27M-mutant diffuse midline glioma (DMG)	NCT04196413
GD2	CAR-T + C7R	high grade glioma (HGG) or diffuse intrinsic pontine glioma (DIPG) or medulloblastoma or another rare brain cancer that expresses GD2	NCT04099797
B7-H3	CAR-T	CNS disease for which there is no standard therapy, or diagnosis of DIPG or DMG at any time point following completion of standard therapy	NCT04185038
IL-13Ra2	CAR-T	Recurrent/Refractory Malignant Brain Tumors	NCT04510051
Her2	CAR-T	recurrent or refractory HER2-positive CNS tumors	NCT03500991
Her2	CAR-T	progressive recurrent or refractory HER2-positive primary central nervous system (CNS) tumor or HER2 positive tumor metastatic to the CNS	NCT02442297
EGFR 806	CAR-T	children and young adults with recurrent or refractory EGFR-positive CNS tumors	NCT03638167

This clinical experience shows that, for all three targets (IL13Rα2, HER2 and CLTX receptor), locoregionally delivered CAR T cells, which are administered on weekly cycles in the outpatient setting without lymphodepletion, have been well-tolerated. Common adverse events include metronomic fevers, headaches, chills and fatigue that occur 24–72 h post each CAR T cell infusion. Concomitant with these toxicities are locoregional increases in inflammatory cytokines, including the IFNγ-responsive cytokines CXCL9 and CXCL10, along with an influx of endogenous immune cell populations detected in the CSF, suggesting that CAR T cell infusions alter the immune landscape. As is often observed with immunotherapies, patient antitumor responses have been mixed; although, encouragingly, many patients have experienced extended stable disease and/or long-term remission that is not typically achieved in the setting of recurrent GBM. One individual with multifocal recurrent GBM achieved complete tumor remission, even though the tumor did not homogenously express IL13Ra2 target antigen [12]. Upon further investigation, it was found that this patient’s tumor had high CD3 infiltrates and, following CAR treatment, increased antitumor T cell reactivity was detected, supporting the notion that CAR T cells may promote host antitumor immunity. To elucidate the mechanism by which this occurs, syngeneic mouse studies have since revealed that CAR T cell IFNγ production activates intratumoral myeloid populations and induces antigen spread [16]. These studies therefore suggest that the interplay between CAR T cells and host immunity is an important consideration for optimizing the activity of CAR therapy in the setting of solid tumors.

CAR T cell therapy is also being evaluated in pediatric brain tumors. For example, GD2, a well-recognized tumor target in several tumor types, is now being evaluated in brain tumors [17, 18]. Stanford recently launched a phase 1 trial using autologous GD2 CAR Ts for diffuse intrinsic pontine glioma (DIPG) and spinal diffuse midline glioma (with assistance from PICI and CureSearch), NCT04196413. All patients with pontine gliomas have an Ommaya placed to allow monitoring of intracranial pressure and management by removing CSF. The trial launched with intravenous (IV) administration following lymphodepleting chemotherapy, but if patients have clinical benefit (stable disease or partial response [PR]), patients may receive subsequent infusions (intracerebral) ventricularly via the Ommaya. In the patients with diffuse myeloid gliomas, anti-tumor effects have been observed after lymphodepletion and IV GD2-CAR T cells. After subsequent intracerebroventricular CAR T infusions without lymphodepletion, the team has observed significant but manageable toxicity (which was well predicted by preclinical models). Thus far, the impressive clinical responses have been followed by recurrence, and the study continues to dose escalate.

Researchers have not observed evidence for GD2 antigen loss and are examining samples obtained to understand the basis for resistance in an attempt to sustain and improve the anti-tumor effects. Everything currently understood about pediatric tumors thus far suggests that they have a lower mutational burden and are less immunogenic than the adult counterparts. In many correlative assays, including in the CSF, many inflammatory markers and impact of the myeloid cells are seen. Some lymphocytes are detected, but many are myeloid cells, which may be modulating the response perhaps in an adverse way.

Although CAR T therapies demonstrated remarkable outcomes in treating hematologic malignancies, the above experience confirms that the development of effective CAR T therapies for solid cancers, including GBM, remains a challenge due to multiple barriers, including the antigenic heterogeneity, on-target off-tumor toxicities, and T cell exhaustion. For example, while a recent CAR study targeting epidermal growth factor receptor variant III (EGFRvIII) [19], safely demonstrated GBM infiltration of IV-infused CAR T cells and reduction of EGFRvIII-positive GBM cells, tumors still recurred, likely due to regrowth of EGFRvIII-negative tumor cells [20].

To date, there are no GBM-specific antigens that are uniformly present in the tumor tissue. On the other hand, while non-mutant glioblastoma-associated antigens (GAAs), such as ephrin type A receptor 2 (EphA2) and IL13Rα2, are more uniformly expressed in GBM, they are also expressed in some non − CNS organs (https://www.proteinatlas.org), raising a concern of off-tumor toxicity outside of CNS. As a way to safely and effectively target GAAs, a novel synthetic Notch “synNotch” receptor system [21] and T-cell circuits that recognize tumor cells based on the “prime-and-kill” strategy have been developed. In this system, the first antigen, which is expressed exclusively on GBM cells (eg, EGFRvIII) or in the brain (e.g., myelin oligodendrocyte glycoprotein [MOG]), primes the T cells to induce expression of a CAR that recognizes EphA2 and IL13Rα2, thereby eradicating GBM cells expressing either EphA2 or IL13Rα2.

These data demonstrates that IV-infused EGFRvIII-synNotch − primed EphA2-/IL13Rα2-CAR T cells are effectively but restrictedly activated by EGFRvIII as the GBM-specific signal, thereby leading to complete eradication of orthotopic patient-derived xenografts (PDXs) with heterogeneous EGFRvIII expression without attacking EphA2/IL-13Rα2-positive cells outside of the CNS. Moreover, a circuit primed by MOG-synNotch also demonstrated effective induction of CAR in the CNS, resulting in persistent remission of intracranial PDX without affecting EphA2/IL-13Rα2-positive cells in the subcutaneous space. Furthermore, these synNotch-CAR T cells were significantly more efficacious than conventional, constitutively expressed CAR T cells, associated with excellent persistence (> 100 days in vivo) and more juvenile (eg, T stem central memory cell) phenotype compared with conventional CAR T cells. Taken together, these data suggest that transcriptional circuits, such as the synNotch system may overcome many of the key challenges in CAR T therapy for solid cancer including off-tumor toxicity, antigen heterogeneity and lack of persistence. Initialclinical testing is anticipated in coming months.

As an alternative to CAR T therapy, the generation and expansion of host antitumor immunity through vaccination is another strategy to generate and expand tumor-specific T-cell responses.

Technological advances have enabled the development of neoantigen cancer vaccines that are tailored to each patient. This approach relies on patient specific genetic alterations in tumors such as single nucleotide variations (SNVs), insertions and deletions (indels), and gene fusions that can lead to the formation of abnormal proteins and mutation-derived tumor antigens called neoantigens. These mutated proteins are degraded in proteasomes. The resulting peptides are transported into major histocompatibility complex (MHC) class I molecules and presented on the surface of tumor cells. This peptide-MHC complex, once recognized by T cells, will trigger an immune response. Exploiting this biological phenomenon of “endogenous vaccine” production provides the rationale to develop immunotherapies like cancer vaccines that encode TAAs individually tailored to each patient and capable of provoking an immune response. The expectations are that once the vaccines are administered to patients, the peptides are processed by DCs. DCs will present the antigens on MHC molecules and prime and activate effector cytotoxic cluster of differentiation (CD)8 + T cells. These cells modify the tumor microenvironment by infiltrating the tumor, recognizing the specific antigens on tumor cells, and eliminating them. High-throughput next-generation whole-exome and ribonucleic acid (RNA) sequencing have allowed the identification of nonsynonymous mutations even in tumors such as GBM, which are generally known for having a low mutation burden. In silico algorithms are used for somatic variant calling, verifying variant expression, neoepitope MHC class I binding affinity prediction and ranking, vaccine peptide selection, and peptide manufacturability determination. Recent trials with shared-non-mutated, shared-mutated, and personalized-mutated antigens in peptide + adjuvant forms have been tested, showing safety, an antigen-specific antibody response [22], sustained memory CD8 + or TH1 CD4 + T cell response against personalized antigen peptides [23] as well as an association between the development of vaccine-specific CD8 + effector memory cells and median OS [24]. More recently, a pilot randomized study with pre-surgical vaccinations with GBM stem cell lysate in patients with WHO grade II low-grade glioma demonstrated homing of vaccine-reactive CD8 + T-cell clones and increased tissue resident-like CD8 + T-cells in the surgically resected glioma following the neoadjuvant vaccination [25].

Another example of collaboration is in the generation of precision vaccines. Bioinformatic pipelines using different computational tools or workflows to identify neoantigens can be potential sources of discrepancy among groups that can reflect different clinical trial results. For example, the OpenVax neoantigen prediction pipeline [26] is used in a current neoantigen vaccine trial for GBM [27]. This pipeline supports SNVs and indel variants, calling somatic variants from tumor and normal deoxyribonucleic acid (DNA) and computing the mutant peptide from corresponding RNA-Seq data. This workflow ranks long synthetic peptides corresponding to each variant by computing a score that combines the prediction of MHC class I affinity and estimation of variant-specific expression [28]. Optimizing the prediction algorithms and converging to a consensus will improve the precision of the prediction and benefit the field. The Parker Institute of Cancer Immunotherapy (PICI) recently led a consortium effort which identified key attributes of immunogenic neoantigens [29] Validation by mass spectrometry may also enhance the accuracy of the prediction. Other relevant caveats and sources of variability in neoantigen predictions include the following: (1) use of fresh tissue (snap frozen) vs formalin-fixed paraffin embedded tissue; (2) variable depth of DNA and RNA sequencing coverage; (3) peptide manufacturing has its complexity, and synthesis can be challenging for some peptide sequences. Peptide folding and conformation can be an obstacle to peptide synthesis that can abrogate immunogenicity, and the execution of purification steps can be another hurdle; (4) vaccine-delivery vehicles and immune adjuvants are also crucial in potentiating immunogenicity; and (5) the turnaround time from the harvest of tumor tissue to peptide synthesis may not apply at the current state of the art for recurrent tumors but is quite feasible for a newly diagnosed GBM. For those patients, the most practical solution is to give off-the-shelf pre-made peptides that target shared recurrent antigens and driver mutations that are known to be present in their specific tumor.

Customized treatment of a patient with multipeptide clinical-grade vaccines is possible, but the costs escalate with the increased number of peptides used to target multiple epitopes. Detailed immune monitoring, including T-cell assays and neoantigen-specific T-cell receptor repertoire, is crucial for evaluating response. As with other technologies, it is conceivable that peptide production becomes a faster process and more affordable. Combination treatment of neoantigen vaccine with other modalities may enhance and potentiate immunotherapy effects for GBM and other brain tumors.

## What is the state of IO in brain tumors in industry?

In addition to traditional vaccines, Biotech companies that are developing brain tumor immunotherapies employ multiple genetic engineering strategies to generate viral or plasmid constructs that target tumor cells while sparing normal neurons, and affect the tumor microenvironment to induce immune responses. As with the academia and nonprofit trials described above, many of these products are delivered intracranially into the tumor cavity to bypass the blood–brain barrier and maximize targeted effect.

The oncolytic adenovirus immunotherapy DNX-2401 (tasadenoturev; DNAtrix, Inc.) is engineered to have a 24 bp deletion for safety plus an RGD-4C insertion for enhanced tumor cell infection. It has demonstrated the ability to induce complete responses in patients with recurrent GBM after a single monotherapy treatment, with evidence of immune changes in the tumor microenvironment post treatment [30]. In a recent phase 2 study of DNX-2401 followed by the PD-1 inhibitor, pembrolizumab (CAPTIVE/KEYNOTE-192), additional clinical benefit was observed in recurrent GBM, including durable responses and further improvement in median overall survival [31].

Inovio’s synthetic DNA-encoded plasmids have demonstrated the ability to create, in combination with cemiplimab (Libtayo^®^, an anti-PD-1 monoclonal antibody from Regeneron/Sanofi) and radiation/chemotherapy, antigen-specific killer T cells directed toward the tumor-associated antigens encoded within the DNA plasmid INO-5401. This is a major first step in being able to potentially defeat tumors that express the antigens for which INO-5401 encodes.

PVSRIPO immunotherapy (Istari Oncology) is based upon the Sabin type 1 polio virus, but with its cognate internal ribosomal entry site machinery replaced by one from the related rhinovirus. This engineering renders PVSRIPO completely incapable of replicating in normal neurons, while retaining its ability to infect and kill the tumor cells. PVSRIPO induces tumor-specific cytotoxic CD8 T-cell responses via activation of antigen-presenting cells such as DCs and tumor-associated microglia that express the poliovirus receptor CD155 on their surface, dramatically increasing interferon secretion and upregulating antigen presentation. [32].

VBI-1901 (VBI) is a DC-type vaccine, designed to trigger an immune response to a highly immunogenic antigen (cytomegalovirus [CMV]) that is present in greater than 90% of GBM tumor specimens. The key considerations in the design of VBI-1901 are (1) boost both CD4 + (CMV gB) and CD8 + (CMV pp65) T-cell responses, (2) elicit a broadly reactive T-cell repertoire against multiple epitopes to avoid rapid tumor immunoselection/escape, and (3) have a potent delivery system where enveloped virus-like particles (eVLPs) expressing the CMV gB and pp65 antigens are formulated with granulocyte–macrophage colony-stimulating factor and given intradermally. In an early 10-patient trial in recurrent GBM, a 40% disease control rate was observed (2 stable disease, 2 PR), which translated to a 12-month overall survival (OS) rate, approximately doubling that of historical controls (NCT03382977) [33].

Imvax’s IGV-001 is an autologous tumor cell vaccine treated ex vivo with insulin-like growth factor type 1 receptor antisense oligonucleotide, encapsulated in biodiffusion chambers, and implanted in the patient’s abdomen. In a small phase 1b study in newly diagnosed GBM patients, the best clinical response as measured by progression-free survival and OS was observed in patients who received the highest dose of IGV-001, particularly those with O^6^-methylguanine-DNA-methyltransferase methylated tumors. These clinical endpoint improvements were accompanied by striking radiographic improvements with sustained meaningful quality of life. [34].

The main characteristics of Oncorus’s genetically engineered oncolytic HSV-1 (oncolytic herpes simplex virus [oHSV]) platform are (1) the retention of HSV ICP34.5 gene that endows viruses with full replication competency and enhanced potency in the face of host antiviral response, (2) a large transgene capacity enabling the tailoring of an optimal payload combination of up to 8 payloads to counteract the immune-suppressive drivers in GBM, and (3) a cell-specific attenuation strategy utilizing the tumor-to-normal cells’ differential expression of microRNAs. The first Oncorus product ONCR-177 for solid tumors with liver metastases is in an early-stage clinical trial (NCT04348916). The company is engineering a microRNA-attenuated armed oHSV drug candidate for GBM.

Still, many hurdles need to be overcome before these approaches turn into successful immunotherapies for GBM. Participants of the workshop stressed the necessity for better mechanistic understanding of the effects of combinations with immune checkpoint blockade; the need for understanding how activation of cytotoxic T cells can generate meaningful clinical responses and prolong OS; the need for peripheral blood biomarker(s) of immune response that would be paired with tumor tissue; the need for better access to post-treatment tumor material from clinical responders; and implications of the high bar for responses in GBM set by response assessment in neuro-oncology (RANO) and immunotherapy response assessment in neuro-oncology (iRANO), which limit sample size for correlating clinical responses to immune activity.

## Preclinical testing considerations

It is recognized that there are certainly limitations with preclinical models, however, such models still aid in understanding mechanisms of action and resistance, as well as testing basic cancer biology and immunobiology. Identification of biomarkers of interest should also be investigated, as they can provide a scientific basis or mechanistic rationale for particular combination approaches. This is crucial, as the mechanism of action plays a key role in selecting specific immunotherapies for combination approaches. Animal models are also critical to safety profile testing, and analysis of observed responses also factor into patient selection algorithms. Preclinical models can be useful for reverse translational studies in which unexpected responses (or lack of response) seen in the clinic can be traced to specific mutations in patients [35]. Preclinical evidence can also help with patient recruitment for trials, especially N of 1 trials. Finally, preclinical data may be necessary for securing funding or for continued development support, although there are inconsistencies in which models are required for positive grant reviews (e.g., some reviewers may accept syngeneic models, while others may require humanized mouse models).

Several challenges with preclinical models were identified during the course of the workshop, and there is a clear need to revisit expectations with preclinical models, which are the current bar for clinical setting evaluation. It is evident that we lack a true representative GBM model. Issues such as molecular drivers, relevant antigens, and the stroma in murine models may be significantly different from those in true GBMs. Furthermore, modeling of tumor heterogeneity can be challenging with preclinical models. This necessitates an increased investment in development and improvement of genetically engineered mouse models (GEMMs) or PDX models.. Lack of standardization with preclinical models is also a limitation (e.g., humanized mice from the same labs may not be similar). Future exploration of other animal models, including spontaneous glioblastoma in canines, is warranted [36].

Organoid models for study of the tumor microenvironment may be used to supplement clinical and murine data. These models may be more representative of a patient’s tumor microenvironment, as they recapitulate some of the features of patient tumors. They are, however, limited in how long they can be kept viable ex vivo. Artificial intelligence (AI) and deep learning may also be used as a complement to preclinical testing to help validate hypotheses. In breast cancer, for example, there has been a recent emergence of models using AI and deep learning to help better predict outcomes. AI models can also be useful in helping identify biomarkers that are more prevalent than others.

## Patient identification and selection considerations

Performing multiomics analyses in a centralized or standardized approach on a subset of patients who had good responses to previous therapies or identified as potential candidates for response can yield important insights and generate novel hypotheses. In-depth analysis of patient subsets can help identify characteristics that lead to improved response. Additionally, selecting a subset of patients may help alleviate certain challenges (particularly for a negative-outcome trial) and expenses of doing such analyses on all the patients. A centralized approach can also help reduce the burden on labs that may lack the infrastructure to conduct such analyses, as well as provide reliable and consistent data.

Although early clinical development can help identify a subset of patients who are good responders, inclusion of an unselected population in a phase 2 or 3 trial can potentially lead to dilution of clinical benefit. Thus, it may be of benefit to delay phase 2 or 3 trials to allow for identification of appropriate patients who are more likely to be responders. Enriching a final trial population with such patients can lead to improved outcomes with immunotherapies for brain tumors. This can further aid in advancing the treatment landscape and re-engage pharmaceutical and biotech companies (as there is a dampening of interest from negative outcomes). Creating infrastructure and platform trials that allow for collaboration toward a common goal (e.g., developing a robust marker panel to help determine optimal patient selection) can also be of benefit.

## Tissue sampling and tissue biopsy considerations

Although blood samples can provide important information, tissue biopsies and CSF sampling provide especially meaningful insights into the local microenvironment where therapies are delivered. Lumbar punctures allow for an increased sample volume (as compared with an Ommaya reservoir); however, serial lumbar punctures can be inconvenient and challenging for patients. On the other hand, the Ommaya reservoir for CSF sampling may be more feasible, well-tolerated, and allows for a time course over therapeutic treatment. However, challenges do exist with an Ommaya reservoir, including increased risk of infection, edema, and herniation.

Earlier sampling of biospecimens (e.g., blood, or CSF) is ideal and provides an actionable baseline; however, appropriate timing for sampling also depends on the treatment modality and response of interest (e.g., persistence and durability of CAR T cells vs. persistence and durability of immune response by DC vaccines). Additionally, early sampling can be challenging for some patients who go directly into surgery. For such patients, a potential baseline may be the time point between surgery and before radiation is received. Challenges with sample collections on multicenter trials (ensuring consistency among centers) as well as outside of clinical trials (e.g., variability among centers) also exist. Data collection and subsequent analysis for identification of ideal timing of sampling is needed.

As compared with other cancers, accessibility of brain tumors for tissue biopsies can be a major barrier. At certain institutions, resistance from clinicians on performing biopsies for patients randomized to the standard of care treatment arm can be challenging. Due to recent advances, tissue biopsy is now standard of care in pediatric DIPG, which may help overcome such challenges.

## Molecular testing considerations

Several advances in molecular testing have been made, yet certain challenges remain. Discordance between immunohistochemistry and molecular analysis for specific mutations can be a hindrance to decision-making. Limited mutation panels for testing at institutional pathology labs, especially community hospitals, can lead to exclusion of specific mutations, which may impact treatment choices. There is a need to inform patients about the potential to send out samples to Foundation Medicine or larger centers with broader panels for a more robust analysis. Lastly, the inability to analyze whole genome sequencing can limit further understanding of such datasets.

Although coverage for isocitrate dehydrogenase mutation testing is a recent development, payers do not cover most molecular testing. The federal government may impact Centers for Medicare & Medicaid Services (CMS) coverage decisions; however, their influence on private health insurer decisions is limited. Potential avenues for overcoming barriers to coverage include conveying to payers the importance of testing for decision making (especially CMS, as private insurers tend to follow CMS decisions) and targeting proprietary companies that private insurances may use to set medical necessity. Certain patient policy advocacy groups like National Brain Tumor Society are also working on legislation to improve coverage for molecular testing.

## Heterogeneity considerations

Tumor heterogeneity is a major concern in GBM; however, approaches to overcoming these challenges do exist. In-depth genomics and immune profiling data from well-designed studies with small sample sizes can be of benefit. These data may help with patient response evaluation and identification of signals, which may indicate if specific immunotherapy is of benefit. However, such analyses may be challenging to conduct in trials with a large patient population.

High-dimensional immune profiling can aid in understanding benefits of immune responses and in identifying tumor subtypes that may be responsive to therapies. Multiplexed ion beam imaging analysis of responder and non-responder tissues and targeting multiple TAAs or tumor-specific mutations, as compared to a single, widely expressed mutation/antigen, can also be valuable. Additionally, analyzing the impact of tumor genetics on the tumor microenvironment can aid in identifying drivers of suppression, which can help guide strategies for therapies with increased likelihood of overcoming suppression.

Inter- and intra-patient heterogeneity necessitates the need for a personalized medicine approach. Identifying baseline tumoral characteristics (e.g., antigen expression) can help improve trial designs, drive optimal patient selection, and potentially identify resistance mechanisms. Additionally, preservation of tissue in a manner that is easy to share is also important for furthering consistent analyses between patients.

## Treatment decision considerations

The following strategies can be helpful in informing future trial designs: analysis of negative trials to better understand certain aspects that may have contributed to the outcomes (i.e., missteps to avoid); avoidance of rushing into phase 2/3 trials and performing correlative analyses to better select populations for subsequent trial designs; and analysis of available data (clinical and preclinical) from other solid tumor trials and leveraging those learnings for improved trial designs.

Investment in workshops where investigators can discuss findings, followed by development of algorithms, as well as sharing of data as early as possible to help others make more informed decisions are areas of need. Additionally, a centralized platform that houses research from various investigators can help future researchers start from a more informed basis. It is important to note that hypotheses generated from retrospective analysis of samples (e.g., from a centralized platform) may be therapy specific. However, such platforms may allow for opportunities to examine overlap across similar therapies and identify potential signals of success or failure (which can be further tested).

Improved outcomes observed with neoadjuvant anti-PD-1 immunotherapy, as compared with adjuvant therapy, in GBM [5] highlight the need for the presence of tumor and a non-suppressive immune system for immunotherapy success. The presence of tumor in the setting of a neoadjuvant approach is thought to allow for immunotherapy-driven priming of tumor for immune system response, leading to increased clinical benefit. It is routine practice for patients undergoing craniotomy for tumor resection to receive corticosteroids in the US, and thus immunosuppressive effects of corticosteroids may also negatively impact immunotherapy benefits. Such results underscore the importance of potential confounding variables or factors impacting the observed results.

The following are also important considerations: neoadjuvant treatment may not be feasible for certain patients who may require emergency resection (e.g., newly diagnosed brain tumor with severe headache, nausea, and vomiting, or seizures); however, there may be an opportunity for neoadjuvant treatment in recurrent disease. Also, longitudinal analysis beyond clinical observations has been beneficial in moving the field forward. Innovative approaches for examining responses over time in patients, albeit small trials, can help identify certain trends and potential biomarkers. Finally, specific antigen − targeting therapies are necessary in GBM, as non-specific tumor approach may lead to autoimmunity against normal brain components.

## Response evaluation considerations

There is a need for better imaging techniques for response evaluation. Although objective response rate can be an ideal measure for treatment response over OS, limitations with imaging, especially differentiating between true progression and pseudoprogression, can be a challenge. At the National Institutes of Health (NIH), several resections for patients with negative outcomes to therapies have shown the presence of immune response without tumor or minimal tumor present (pseudoprogression). Misinterpretation of pseudoprogression as true progression can negatively impact outcomes, including discontinuation of effective therapy, a switch to another therapy (which may not be effective), and potential for compromising positive treatment results. On the other hand, misinterpretation of true progression as pseudoprogression can lead to continuation of ineffective therapy, leading to tumor growth and potential transition of care to hospice if eventually deemed untreatable. Furthermore, challenges with imaging may be contributing to the misconception that immunotherapy is not an effective GBM treatment. Unfortunately, serial biopsies are not considered practical for the evaluation of therapies for trials, as all patients may not be appropriate candidates for second resection or multiple resections. This fact further underscores the need for effective imaging and biomarkers that accurately reflect tumor activity vs. treatment effect or a combination of both.

Matched cohorts from a registry or a synthetic control arm may be of assistance in defining responders vs. non-responders more effectively, as compared with change over time from baseline. However, challenges with permission to access and share such data given privacy regulations can be a hindrance. Patient-driven data gathering (e.g., Brain Tumor Project by Count Me In) may be a potential solution, although barriers to data for deceased patients may still persist.

Challenges with criteria for RANO and iRANO as compared with Response Evaluation Criteria In Solid Tumors in stratifying clinical responders (e.g., patients with a low response but who do not meet full response criteria may be classified as non-responders), as well as lack of criterion to help clinicians decide whether to continue treatment or not, also remain. Revising the criterion and investigating new magnetic resonance imaging sequences [36] or other types of imaging, including positron emission tomography, that better determine the changes in the tumor region are needed.

## Patient perspective considerations

It is a misconception that patients are not knowledgeable, given the advent of social media and online message boards, which allow patients to effectively share their knowledge and experience. Partnering with patients in treatment decision making (shared decision making), including review of evidence from preclinical models (which may help boost patient morale or help them stay on treatment), and for clinical trial designs, including endpoints of interest, can be important from the patient’s perspective.

Providing adequate information that is tailored to patients’ level of understanding and empowering them to make informed decisions is vital. Additionally, comprehensive information, including current and subsequent options (trials and available therapies), and therapy expectations (responses, safety profile, and quality of life implications) should be shared with patients. Quality of life measures, including emotional, social, physical, and cognitive functioning are valued by patients.

Access to experimental drugs for N of 1 trials and omics analysis, inclusion of multiple brain tumor subtypes in clinical trials, and access to high-level summary of the proposed white paper for patients and caregivers are also meaningful to patients. Although patients do see the importance of clinical trials and research, interest in participation may vary depending on the stage of the patient’s journey.

## Conclusions and future directions

Several unmet needs exist in brain tumors, including exploring avenues to help bring IO to the newly diagnosed setting (current research and work is primarily in the recurrent disease setting); identification of putative markers or biomarkers that will help identify which patients are more (or less) likely to respond to immunotherapy (ie, responders vs non-responders); access to therapy (including experimental drugs) for subgroups of patients who may derive benefit from them (eg, N of 1 trial); and research to help identify patient factors that render immunotherapy less effective in certain patients. In-depth profiling of biopsies may yield such insights. Another need is a unique data platform that allows for (a) integration of data from various models (preclinical, clinical, and organoid) and datasets, (b) retrospective analysis of data to help inform future trial designs, (c) functionality for input of data from various users (comprehensive datasets), and (d) development of an algorithm (AI learning) based on the available data to help guide decisions.

Interaction between the CNS and the bone marrow in GBM is an understudied area. Better understanding of the life cycle of various myeloid cell compartment constituents from early progenitor cells to differentiation is needed (e.g., predetermined differentiation or factors influencing cell differentiation at the final destination; cell cycle stage in bone marrow vs. blood vs. CSF, etc.). Identifying trends in myeloid cell skewing (e.g., recognizing the absence of a specific myeloid cell type as opposed to what is present), as well as the effect of radiation and chemotherapy on the myeloid cell compartment also needs further research. Caution should be exercised when examining blood samples to understand the cell’s activity in the brain.

Examining immunotherapy combinations (e.g., combinations with other IO, neoadjuvant/adjuvant therapy, radiation, chemotherapy, DNA damage inhibitors, targeted therapies, vaccines, cellular therapies, etc.) in smaller trials is necessary before implementation in larger platforms such as GBM AGILE [37]. Caution with positive findings or signals from small trials should be exerted, as larger well-powered trials may be necessary to determine full clinical impact of the findings. A smaller platform trial (n = 15) with deep immune profiling is an important approach to consider [38].

## Data Availability

Not applicable.

## Abbreviations

AI: Artificial intelligence
CAR T: Chimeric antigen receptor T cell
CD: Cluster of differentiation
CMS: Centers for Medicare & Medicaid Services
CMV: Cytomegalovirus
CNS: Central nervous system
CSF: Cerebrospinal fluid
DC: Dendritic cell
DIPG: Diffuse intrinsic pontine glioma
EGFRvIII: Epidermal growth factor receptor variant III
EphA2: Ephrin type A receptor 2
GAA: Glioblastoma-associated antigen
GBM: Glioblastoma
GEMM: Genetically engineered mouse model
IV: Intravenous
IL13R: Interleukin-13 receptor
IO: Immuno-oncology
iRANO: Immunotherapy response assessment in neuro-oncology
MHC: Major histocompatibility complex
MOG: Myelin oligodendrocyte glycoprotein
NBTS: National Brain Tumor Society
oHSV: Oncolytic herpes simplex virus
OS: Overall survival
PD-1: Programmed death-1
PDX: Patient-derived xenograft
PICI: Parker Institute for Cancer Immunotherapy
PR: Partial response
RANO: Response assessment in neuro-oncology
SNV: Single nucleotide variations
TAA: Tumor associated antigens

## References

[1] Chuntova P, Chow F, Watchmaker PB (2021). Unique challenges for glioblastoma immunotherapy—discussions across neuro-oncology and non-neuro-oncology experts in cancer immunology. Meeting Report from the 2019 SNO Immuno-Oncology Think Tank. Neuro-Oncology.

[2] Khasraw M, Reardon DA, Weller M, Sampson JH (2020). Do they have a future in the treatment of Glioblastoma. Clin Cancer Res.

[3] Cloughesy TF, Mochizuki AY, Orpilla JR (2019). Neoadjuvant anti-PD-1 immunotherapy promotes a survival benefit with intratumoral and systemic immune responses in recurrent glioblastoma. Nat Med.

[4] Müller S, Kohanbash G, Liu SJ (2017). Single-cell profiling of human gliomas reveals macrophage ontogeny as a basis for regional differences in macrophage activation in the tumor microenvironment. Genome Biol.

[5] Cloughesy TF, Mochizuki AY, Orpilla JR (2019). Neoadjuvant anti-PD-1 immunotherapy promotes a survival benefit with intratumoral and systemic immune responses in recurrent glioblastoma. Nat Med.

[6] Lu Y, Ng AHC, Chow FE (2021). Resolution of tissue signatures of therapy response in patients with recurrent GBM treated with neoadjuvant anti-PD1. Nat Commun.

[7] Keren L, Bosse M, Marquez D (2018). A structured tumor-immune microenvironment in triple negative breast cancer revealed by multiplexed ion beam imaging. Cell.

[8] Keren L, Bosse M, Thompson S (2019). MIBI-TOF: a multiplexed imaging platform relates cellular phenotypes and tissue structure. Sci Adv.

[9] Brown C, Aguilar B, Starr R (2018). Optimization of IL13Rα2-targeted chimeric antigen receptor T cells for improved anti-tumor efficacy against glioblastoma. Mol Ther.

[10] Priceman SJ, Tilakawardane D, Jeang B (2018). Regional delivery of chimeric antigen receptor-engineered t cells effectively targets HER2 + breast cancer metastasis to the brain. Clin Cancer Res.

[11] Theruvath J, Sotillo E, Mount C (2020). Locoregionally administered B7–H3-targeted CAR T cells for treatment of atypical teratoid/rhabdoid tumors. Nat Med.

[12] Brown C, Alizadeh D, Starr R (2016). regression of glioblastoma after chimeric antigen receptor T-cell therapy. N Engl J Med.

[13] Johnsson VD, Ng RH, Dullerud R (2021). CAR T cell therapy drives endogenous locoregional T cell dynamics in a responding patient with glioblastoma. bioRxiv.

[14] Portnow J, Wang D, Blanchard MS (2020). Systemic anti-PD-1 immunotherapy results in PD-1 blockade on T cells in the cerebrospinal fluid. JAMA Oncol.

[15] Wang D, Starr R, Chang WC (2020). Chlorotoxin-directed CAR T cells for specific and effective targeting of glioblastoma. Sci Transl Med.

[16] Alizadeh D, Wong R, Gholamin S (2021). IFNγ Is critical for CAR T cell-mediated myeloid activation and induction of endogenous immunity. Cancer Discov.

[17] Mount CW, Majzner RG, Sundaresh S (2018). Potent antitumor efficacy of anti-GD2 CAR T cells in H3–K27M^+^ diffuse midline gliomas. Nat Med.

[18] Long AH, Highfill SL, Cui Y (2016). Reduction of MDSCs with all-trans retinoic acid Improves CAR therapy efficacy for sarcomas. Cancer Immunol Res.

[19] Autologous T cells redirected to EGFRVIII-with a chimeric antigen receptor in patients with EGFRVIII+ glioblastoma. ClinicalTrials.gov identifier: NCT02209376. Updated March 5, 2019. https://clinicaltrials.gov/ct2/show/NCT02209376. Accessed 1 Sept 2021.

[20] O'Rourke DM, Nasrallah MP, Desai A (2017). A single dose of peripherally infused EGFRvIII-directed CAR T cells mediates antigen loss and induces adaptive resistance in patients with recurrent glioblastoma. Sci Transl Med.

[21] Choe JH, Watchmaker PB, Simic MS (2021). SynNotch-CAR T cells overcome challenges of specificity, heterogeneity, and persistence in treating glioblastoma. Sci Transl Med.

[22] Weller M, Butowski N, Tran DD (2017). for theACT IV trial investigators, Rindopepimut with temozolomide for patients with newly diagnosed, EGFRvIII-expressing glioblastoma (ACT IV): a randomised, double-blind, international phase 3 trial. Lancet Oncology.

[23] Hilf N, Kuttruff-Coqui S, Frenzel K (2017). Actively personalized vaccination trial for newly diagnosed glioblastoma. Nature.

[24] Mueller S, Taitt JM, Villanueva-Meyer JE (2020). Mass cytometry detects H33K27M-specific vaccine responses in diffuse midline glioma. J Clin Invest.

[25] Ogino H, Taylor JW, Nejo T (2022). Randomized trial of neoadjuvant vaccination with tumor-cell lysate induces T cell response in low-grade gliomas. J Clin Invest.

[26] Kodysh J, Rubinsteyn A (2020). OpenVax: an open-source computational pipeline for cancer neoantigen prediction. Methods Mol Biol.

[27] Safety and immunogenicity of personalized genomic vaccine and tumor treating fields (TTFields) to treat glioblastoma. ClinicalTrials.gov identifier: NCT03223103. Updated July 8, 2021. https://clinicaltrials.gov/ct2/show/NCT03223103. Accessed 1 Sept 2021.

[28] Boegel S, Castle JC, Kodysh J (2019). Bioinformatic methods for cancer neoantigen prediction. Prog Mol Biol Transl Sci.

[29] Wells DK, van Buuren MM, Dang KK (2020). Key parameters of tumor epitope immunogenicity revealed through a consortium approach improve neoantigen prediction. Cell.

[30] Lang F, Conrad C, Gomez-Manzano C (2018). Phase I Study of DNX-2401 (Delta-24-RGD) oncolytic adenovirus: replication and immunotherapeutic effects in recurrent malignant glioma. J Clin Oncol.

[31] Zadeh G, Daras M, Cloughesy TF (2020). LTBK-04. Phase 2 Multicenter study of the oncolytic adenovirus DNX-2401 (tasadenoturev) in combination with pembrolizumab for recurrent glioblastoma; Captive Study (KEYNOTE-192). Neuro-Oncology.

[32] Gromeier M, Brown MC, Zhang G (2021). Very low mutation burden is a feature of inflamed recurrent glioblastomas responsive to cancer immunotherapy. Nat Commun.

[33] T Berthoud, F Deonarine, S Ng Cheong Chung, C Soare, F Diaz-Mitoma, DE Anderson. CMV-specific immuno-dysregulation in recurrent glioblastoma patients can be overcome with therapeutic vaccination which is associated with tumor response and overall survival benefits in a Phase I/IIa study. ASCO 2020

[34] Andrews DW, Judy KD, Scott CB (2021). Phase Ib clinical trial of igv-001 for patients with newly diagnosed glioblastoma. Clin Cancer Res.

[35] Isakson SH, Rizzardi AE, Coutts AW (2018). Genetically engineered minipigs model the major clinical features of human neurofibromatosis type 1. Commun Biol.

[36] Song J, Kadaba P, Kravitz A, Hormigo A, Friedman J, Belani P, Hadjipanayis C, Ellingson BM, Nael K (2020). Multiparametric MRI for early identification of therapeutic response in recurrent glioblastoma treated with immune checkpoint inhibitors. Neuro Oncol.

[37] A trial to evaluate multiple regimens in newly diagnosed and recurrent glioblastoma (GBM AGILE). ClinicalTrials.gov identifier: NCT03970447. Updated August 20, 2021. https://clinicaltrials.gov/ct2/show/NCT03970447. Accessed 1 Sept 2021.

[38] Stewart MD, Keane A, Butterfield LH (2020). Accelerating the development of innovative cellular therapy products for the treatment of cancer Meeting Report. Cytotherapy.

